# Molecular Characterization of Endometrial Cancer Using a Laboratory-Developed Multi-Gene NGS Panel

**DOI:** 10.3390/ijms27167384

**Published:** 2026-08-18

**Authors:** Thais Maloberti, Annalisa Altimari, Elisa Gruppioni, Laura Poppi, Viviana Sanza, Alessia Costantino, Sara Coluccelli, Giulia Calafato, Floriana Jessica Di Paola, Caterina Ravaioli, Angelo Gianluca Corradini, Marco Grillini, Anna Myriam Perrone, Pierandrea De Iaco, Daniela Rubino, Claudio Zamagni, Giovanni Tallini, Antonio De Leo, Dario de Biase

**Affiliations:** 1 Solid Tumor Molecular Pathology Laboratory, IRCCS Azienda Ospedaliero—Universitaria di Bologna, 40138 Bologna, Italy; thais.maloberti2@unibo.it (T.M.); annalisa.altimari@aosp.bo.it (A.A.); elisa.gruppioni@aosp.bo.it (E.G.); laura.poppi@aosp.bo.it (L.P.); viviana.sanza@ausl.bo.it (V.S.); alessia.costantino@aosp.bo.it (A.C.); sara.colucelli@aosp.bo.it (S.C.); antonio.deleo@unibo.it (A.D.L.); dario.debiase@unibo.it (D.d.B.); 2 Biobank of Research, IRCCS Azienda Ospedaliero—Universitaria di Bologna, 40138 Bologna, Italy; giulia.calafato@aosp.bo.it (G.C.); florianajessica.dipaola@aosp.bo.it (F.J.D.P.); 3 Pathology Unit, IRCCS Azienda Ospedaliero—Universitaria di Bologna, 40138 Bologna, Italy; caterina.ravaioli2@unibo.it (C.R.); angelo.corradini@aosp.bo.it (A.G.C.); marco.grillini@aosp.bo.it (M.G.); 4 Department of Medical and Surgical Sciences (DIMEC), University of Bologna, 40138 Bologna, Italy; myriam.perrone@unibo.it (A.M.P.); pierandrea.deiaco@unibo.it (P.D.I.); 5 Division of Oncologic Gynecology, IRCCS Azienda Ospedaliero—Universitaria di Bologna, 40138 Bologna, Italy; 6 Addarii Medical Oncology, IRCCS Azienda Ospedaliero—Universitaria Di Bologna, 40138 Bologna, Italy; daniela.rubino@aosp.bo.it (D.R.); claudio.zamagni@aosp.bo.it (C.Z.); 7 Department of Pharmacy and Biotechnology (FaBiT), University of Bologna, 40127 Bologna, Italy

**Keywords:** endometrial cancer, molecular characterization, next-generation sequencing, mutations, molecular sub-groups

## Abstract

Endometrial carcinoma is a biologically and clinically heterogeneous neoplasm, for which molecular classification now plays a central role in determining prognosis and guiding treatment. The aim of this study was to develop and apply an in-house multi-gene NGS panel to expand the molecular characterization of endometrial tumors beyond the markers required for surrogate classification. Seventy primary endometrial carcinomas were selected and analyzed by immunohistochemistry for p53, mismatch repair proteins, *ARID1A*, and *PTEN*, and by NGS on DNA extracted from FFPE samples using a panel comprising 28 genes. Molecular classification was assigned according to the WHO algorithm into the subgroups *POLE*-mutated, MMR-deficient, p53-abnormal, and NSMP (No Specific Molecular Profile). Sixty-eight cases were evaluable by sequencing, and in 93% at least one pathogenic, likely pathogenic, or variant of uncertain significance was identified. The most frequent alterations involved *PTEN*, *PIK3CA*, *ARID1A*, and *TP53*. *POLE*-mutated tumors showed the highest mutational burden, while *CTNNB1* alterations were predominantly associated with the NSMP subgroup. Overall, the proposed panel proved to be a useful tool for complementing conventional molecular classification and for highlighting additional genomic differences that may be biologically and clinically relevant.

## 1. Introduction

Endometrial carcinoma (EC) is the most common malignant neoplasm of the female genital tract in Western countries, with an incidence that continues to show a worrying upward trend [1], linked in part to the aging of the population and the prevalence of metabolic factors such as obesity. For decades, the clinical management of these patients has been based almost exclusively on conventional clinico-pathological parameters, such as histotype, grade, and FIGO stage. However, this traditional classification has often shown significant limitations in predicting clinical outcome, failing to capture the intrinsic biological heterogeneity of the disease [1,2]. The diagnostic and therapeutic approach to endometrial carcinoma changed profoundly with the publication of The Cancer Genome Atlas (TCGA) in 2013, which redefined the disease as a molecularly heterogeneous entity. TCGA identified four genomic groups with distinct biological and prognostic features [2,3,4]:

- *POLE*-ultramutated, characterized by pathogenic mutations in the exonuclease domain of the DNA polymerase epsilon gene and associated with an excellent prognosis;
- MSI-H (Microsatellite instability high)-hypermutated, largely corresponding to mismatch repair-deficient tumors, with intermediate prognosis;
- Copy-number high/serous-like, typically associated with *TP53* abnormalities, extensive somatic copy-number alterations, and poor clinical outcome;
- Copy-number low, which largely overlaps with the no specific molecular profile subgroup and usually shows intermediate prognosis.

Since the original TCGA classification relied on complex multi-omics analyses, subsequent studies translated this framework into a clinically feasible surrogate molecular classification based on *POLE* mutation status, mismatch repair status, and p53 expression, identifying four practical groups: *POLE*-mutated (*POLE*), mismatch repair-deficient (MMRd), p53-abnormal (p53 abn), and no specific molecular profile (NSMP) tumors. This integrated molecular approach has been incorporated into international guidelines, including the ESGO/ESTRO/ESP risk classification, and now represents a cornerstone of prognostic stratification and adjuvant treatment decision-making in endometrial carcinoma [5,6,7]. Although immunohistochemistry (IHC) for mismatch repair proteins and p53 can serve as a surrogate for three of the four subtypes, determining the mutational status of *POLE* necessarily requires DNA analysis. In this context, Next-Generation Sequencing (NGS) emerges as the key enabling technology [6,8,9,10,11,12,13,14,15].

Comprehensive commercial genomic profiling assays—such as FoundationOne CDx (324 genes, FDA-approved companion diagnostic), MSK-IMPACT (up to ~500 genes), and TruSight Oncology 500 (TSO500; 523 genes)—provide broad, pan-cancer molecular characterization (Table 1). However, their breadth entails high per-sample cost, substantial bioinformatic and regulatory infrastructure, and, in many settings, reliance on centralized or send-out testing, with consequently longer turnaround and limited local availability. For the molecular classification of endometrial carcinoma, only a defined set of alterations is strictly required—such as *POLE* or *TP53*—together with a limited number of additional genes of established biological or therapeutic relevance. In this context, a focused, laboratory-developed panel that can be run in-house on routinely available FFPE material represents a useful tool, delivering the information needed for guideline-based classification while optimizing cost, tissue use, and turnaround time. The present study was designed to demonstrate the feasibility of such a panel for routine clinical use.

The aim of this study was to develop and clinically validate a focused, laboratory-developed multi-gene NGS panel for the molecular classification of endometrial carcinoma in routine practice, enabling the analysis not only of *POLE* and *TP53* but also of additional genes that may help characterise molecular subgroups, particularly NSMP tumours.

## 2. Results

The study included 70 samples of endometrial carcinoma, and the average age of the patients was 66 years (Table 2 and Table S1). Sequencing quality metrics and read-depth criteria are detailed in Section 4.

With regard to histology, 52 cases are endometrioid carcinomas (76%), 6 are dedifferentiated/undifferentiated carcinomas (10%), 4 are clear cell carcinomas (6%), 3 are carcinosarcomas (4%), and 3 are serous carcinomas (4%). Deficiencies in the mismatch repair system (MMRd) were found in only 25% of cases, with 88% of these cases resulting from a loss of expression of the MLH1 and PMS2 proteins. Following the 2020 WHO algorithm, the cohort was stratified into molecular subgroups as follows: 15 (22%) MMRd group, 16 (22%) p53abn group, 8 (12%) *POLE* group, and 29 (43%) NSMP group.

### 2.1. Next-Generation Sequencing

**Figure 1:**
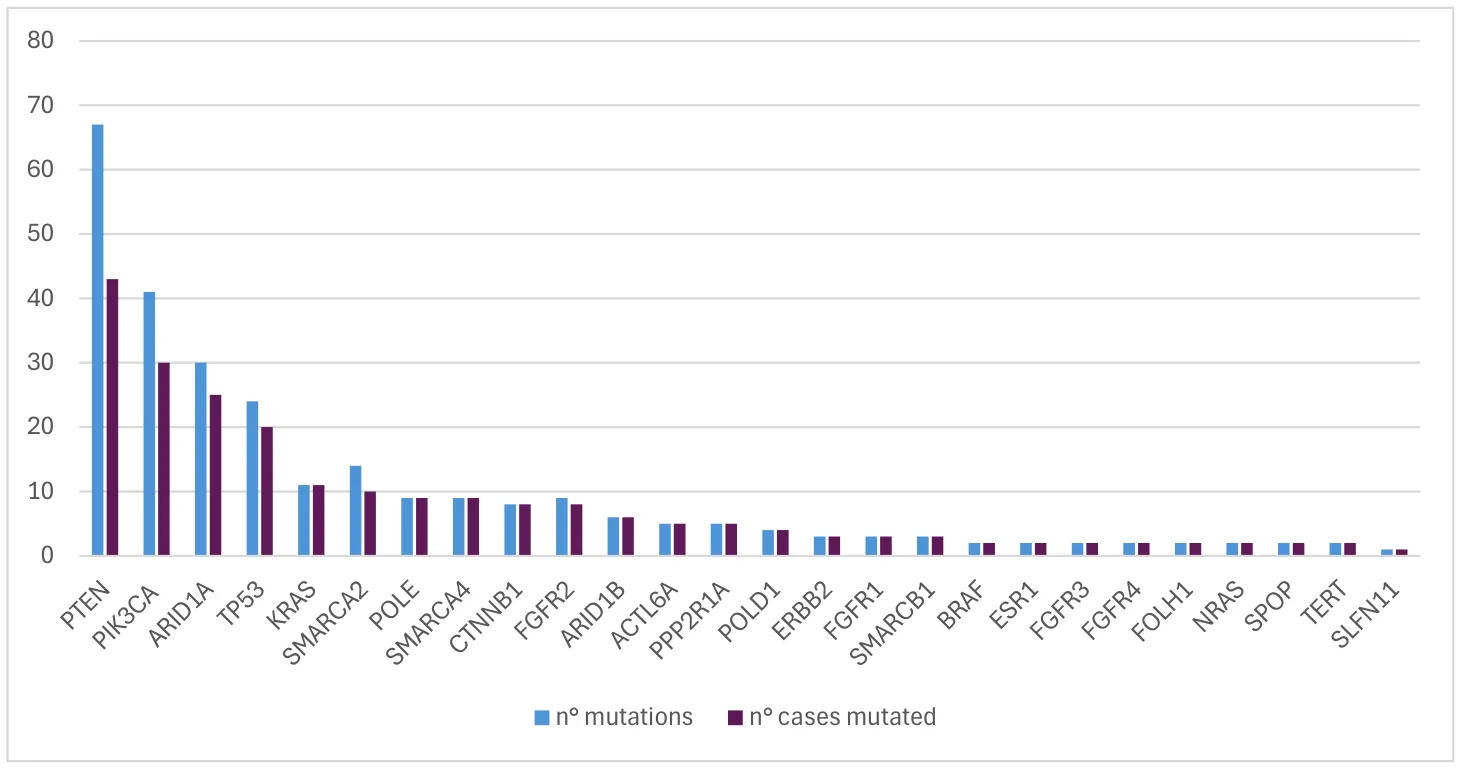
Number of mutations and number of mutated cases in the analyzed cohort. No alteration has been found in *ACTL6B* and *HRAS*.

All 70 samples were evaluated for NGS analysis. In two cases, analysis was not evaluable due to low-quality, low-quantity DNA. In 5 cases (7%), no alterations were detected in any of the analyzed genes. In the remaining 65 cases (93%), 268 pathogenic (P), likely pathogenic (LP), or variant of unknown significance (VUS) variants were identified in 26 genes (Figure 1). Benign or likely benign variants were not observed.

**Figure 2:**
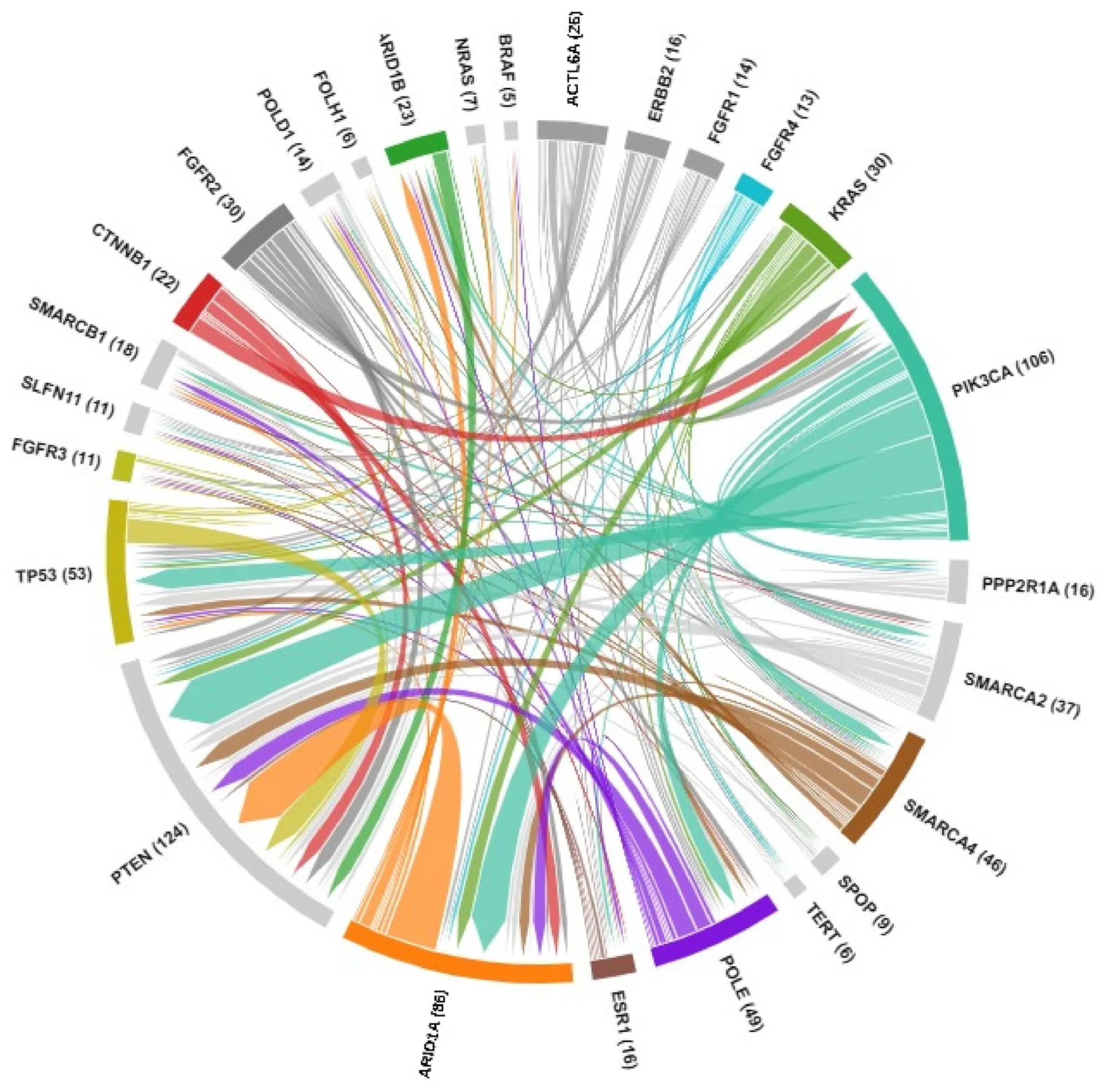
Co-mutations present in our cohort. The thickness of the lines reflects the number of co-mutated samples.

**Figure 3:**
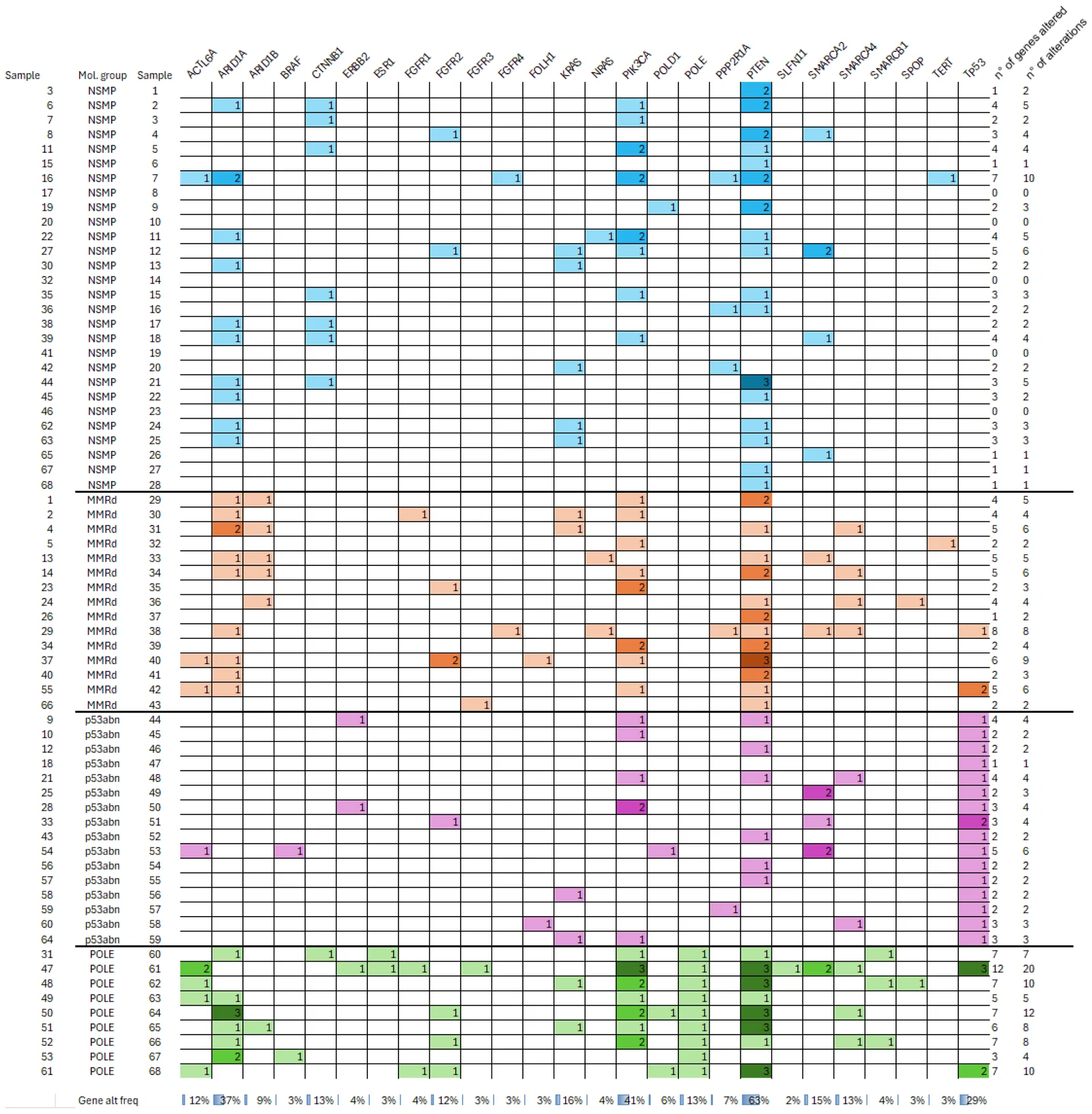
Molecular alteration of each sample, divided by molecular subgroups. For each individual case, the complete set of gene alterations detected is arranged by molecular subgroup. The per-case mutational load and the pattern of co-occurring alterations that characterize each subgroup, rather than the aggregate frequencies, are shown in Figure 5. The boxes are more intensely colored as the number of mutations identified in that gene increases.

The most frequently mutated gene is *PTEN* (in 43% of cases), followed by *PIK3CA* (30%), *ARID1A* (25%), and *TP53* (20%). These genes are also the ones with the highest number of multiple alterations in the same sample. There are several co-mutations (Figure 2 and Figure 3), and the most common ones are as follows: in 21 cases, *PTEN* and *PIK3CA*; in 20 cases, *PTEN* and *ARID1A*; in 15 cases, *ARID1A* and *PIK3CA*; and in 10 cases, *PTEN* and *TP53* co-mutations.

When examining molecular subgroups, the cases with the highest number of mutations are *POLE*-mutated, with an average of 9.3 alterations per sample, ranging from 4 to 20. MMRd cases present an average of 4.6 alterations per case, followed by p53abn cases with 2.9 alterations and NSMP cases with an average of 2.7 alterations per sample. *POLE*-mutated cases are those with the highest rate of multiple alterations in the same gene.

**Figure 4:**
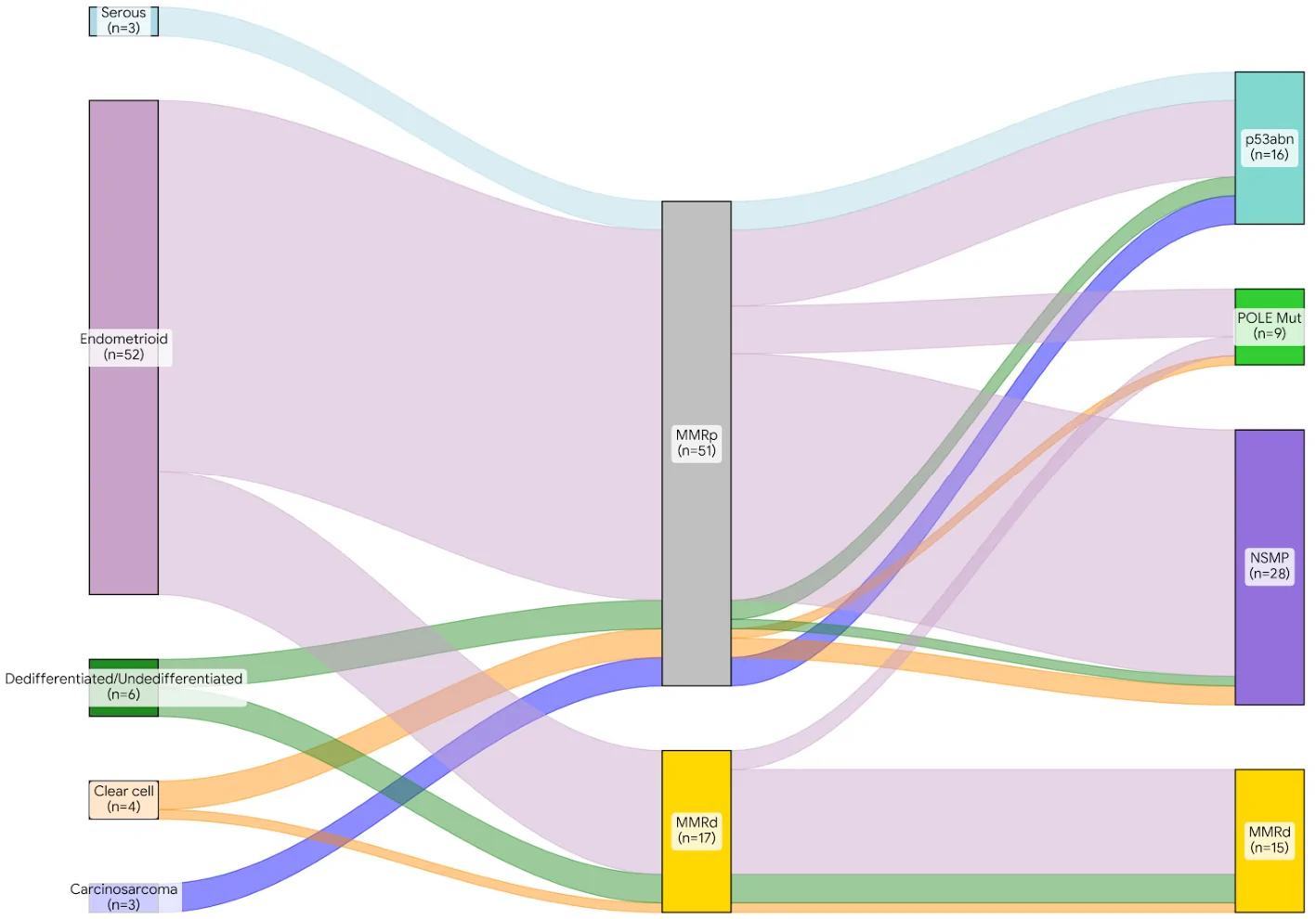
Alluvional plot showing the correlations between diagnosis, mismatch repair status, and molecular subgroup.

Correlating diagnoses with MMR status and molecular subgroup (Figure 4), serous endometrial carcinomas and carcinosarcomas are all MMRp and belong to the p53abn group; dedifferentiated/undifferentiated carcinomas are divided into MMRp tumors, which belong to either the p53abn or NSMP subgroups, and MMRd tumors, which belong to their respective molecular subgroups. MMRd clear cell endometrial carcinomas belong to the same molecular subgroup, while MMRp clear cell endometrial carcinomas are divided into *POLE*-mutated and NSMP. The most numerous group, namely endometrioid ECs, is found in every molecular subgroup.

**Figure 5:**
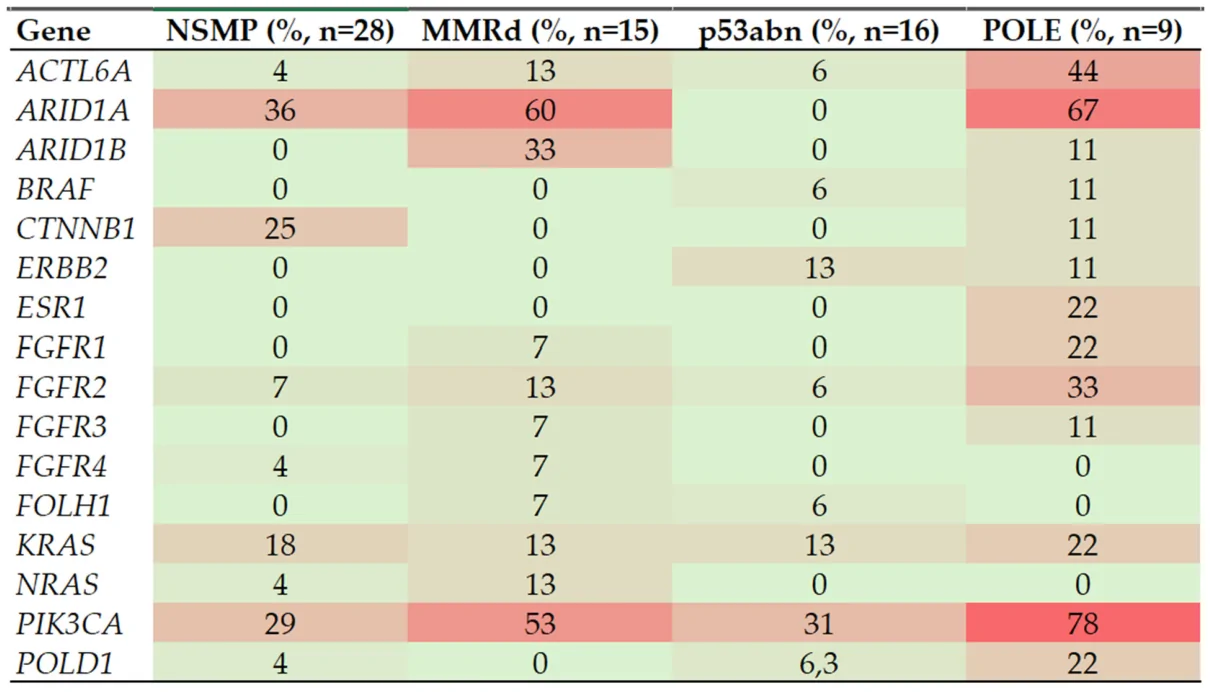
Frequency of the most frequent gene-detected mutations in 4 different molecular subgroups. Green: lower frequency; Red: higher frequency. Only the 16 genes most frequently altered across the cohort are displayed; genes altered in isolated cases were omitted for readability.

The NSMP subgroup showed a heterogeneous mutational profile (Figure 5). The most frequent alterations in this group involved *PTEN*, observed in 17/28 cases (60.7%), followed by *ARID1A* in 10/28 cases (35.7%), *PIK3CA* and *CTNNB1*, each detected in 7/28 cases (25.0%), and *KRAS* in 5/28 cases (17.9%). Alterations in *SMARCA2* were present in 4/28 cases (14.3%).

The MMRd subgroup exhibited a broader, more dispersed mutational spectrum (Figure 5). *PTEN* alterations were present in 12/15 cases, 80.0%, while *ARID1A* was altered in 9/15 cases, 60.0%, and *PIK3CA* in 8/15 cases, 53.3%. *ARID1B* alterations were relatively frequent in this subgroup, occurring in 5/15 cases (33.3%), whereas *SMARCA4* alterations were observed in 4/15 cases (26.7%). Less frequent alterations included *KRAS*, *NRAS*, *ACTL6A*, *FGFR2*, and *SMARCA2*.

The p53abn subgroup was obviously dominated by *TP53* alterations, detected in 16/16 cases (100%) (Figure 5). Additional genomic events were less frequent and more heterogeneous. *PTEN* alterations were observed in 6/16 cases, 37.5%, *PIK3CA* in 5/16 cases, 31.2%, *SMARCA2* in 3/16 cases, 18.8%, and *ERBB2* and *SMARCA4* each in 2/16 cases, 12.5%. Notably, *ARID1A*, *ARID1B*, and *CTNNB1* alterations were not detected in the p53abn subgroup.

The POLE-mutated subgroup showed the highest density of concomitant genomic alterations (Figure 5). In addition to *POLE* alterations, detected in 9/9 cases (100%), frequent co-alterations included *PTEN* in 8/9 cases (88.9%), *PIK3CA* in 7/9 cases (77.8%), and *ARID1A* in 6/9 cases (66.7%). Additional recurrent alterations included ACTGα in 4/9 cases (44.4%), *FGFR2*, *SMARCA4*, and *SMARCB1*, each in 3/9 cases (33.3%), and *ERBB2*, *ESR1*, *FGFR1*, *KRAS*, *POLD1*, and *TP53*, each in 2/9 cases (22.2%).

Among non-class-defining alterations, *PTEN* and *PIK3CA* were broadly distributed across all molecular subgroups and therefore did not show molecular class specificity (Figure 5). In contrast, *CTNNB1* alterations were strongly enriched in the NSMP subgroup, detected in 7/28 NSMP tumors (25.0%) and in only 1/40 (2.5%) non-NSMP tumors (Figure 5). *ARID1B* alterations were mainly observed in MMRd tumors, with only one additional altered case in the *POLE*-mutated subgroup. *ARID1A* alterations were frequent in NSMP, MMRd, and *POLE*-mutated tumors, but were absent in p53abn carcinomas.

A limited number of genes appeared restricted to a single molecular subgroup. Excluding the class-defining genes *TP53* and *POLE*, alterations in *SMARCB1*, *ESR1*, and *SLFN11* were observed exclusively in *POLE*-mutated tumors, occurring in 3/9 (33.3%), 2/9 (22.2%), and 1/9 (11.1%) of cases, respectively. Although these findings should be interpreted with caution, given the small number of *POLE*-mutated tumors, they further support the high mutational burden and distinctive genomic profile of this subgroup.

### 2.2. Comparison of IHC and NGS

The aim of this comparison was to assess the degree of concordance between protein expression evaluated by IHC and the corresponding gene status determined by NGS, and to explore the biological and technical mechanisms underlying eventual discordant cases. IHC was not used as a reference standard to validate the sequencing results, since the two techniques interrogate different molecular levels—protein expression versus DNA sequence—and a lack of agreement does not necessarily reflect a sequencing error. The comparison between the immunohistochemical and NGS data yielded the following results (Supplementary Table S1). Regarding p53, among the 67 cases analyzed, discordance between immunohistochemical findings and *TP53* gene status was observed in only 5 cases (7%). Specifically, one case showed abnormal p53 expression by IHC despite a wild-type (WT) *TP53* profile on NGS, whereas 3 cases were classified as WT by IHC but harbored *TP53* mutations on NGS analysis (p.Arg248Gln, p.Glu286Lys, and p.Val217Met). In addition, one case classified as WT by IHC carried a variant of uncertain significance (VUS) in *TP53* (p.Arg283His).

For ARID1A, both NGS and IHC data were available for 42 cases, of which 12 (28%) showed discordant findings (Supplementary Table S1). In detail, 6 cases exhibited loss of expression by IHC while remaining WT by NGS, one case displayed a subclonal staining pattern by IHC with WT status on NGS, and 5 cases were classified as WT by IHC but harbored mutations on NGS, including two VUS variants (p.Ala2065Pro and p.Arg1989Gln) and three LP/P alterations (p.Glu896Ter, p.Gln1212Ter, and p.Glu2145Asp).

With respect to PTEN, data from both NGS and IHC were available for 50 cases, of which 9 (18%) showed discordant results (Supplementary Table S1). Three cases demonstrated loss of expression by IHC despite WT status on NGS, while 6 cases were classified as WT by IHC but harbored the following LP/P alterations on NGS: p.Glu299Ter, p.Arg130Gln, p.Glu242Ter, p.Tyr46Ter, and p.Arg142Trp.

## 3. Discussion

The molecular characterization of endometrial carcinoma has become an essential component of contemporary diagnostic practice, allowing the integration of morphology, immunohistochemistry, and mutational analysis into a clinically meaningful classification. The surrogate molecular algorithm based on *POLE* mutation status, mismatch repair protein expression, and p53 immunohistochemistry has enabled the routine identification of four major molecular subgroups: *POLE*-mutated, MMRd, p53abn, and NSMP tumors. These groups are associated with distinct biological and prognostic features and have been incorporated into current risk stratification systems and therapeutic decision-making. However, while this classification represents a major advance, it does not fully capture the genomic complexity of endometrial carcinoma, particularly within the NSMP category. In this study, we applied a customized multi-gene NGS panel to characterize both class-defining and additional genomic alterations across molecular subgroups of endometrial carcinoma.

Overall, our findings confirm the feasibility and utility of an extended NGS-based approach in the molecular work-up of endometrial carcinoma. In addition to detecting *POLE* and *TP53* alterations, which are directly relevant for molecular classification, the panel identified a broad spectrum of additional genomic events involving genes related to the PI3K/AKT/mTOR pathway, Wnt/β-catenin signaling, chromatin remodeling, receptor tyrosine kinase pathways, and RAS/MAPK signaling. The most frequently altered genes in the overall cohort were *PTEN*, *PIK3CA*, *ARID1A*, and *TP53*, in keeping with the known molecular landscape of endometrial carcinoma. *PTEN* and *PIK3CA* alterations were widely distributed across molecular subgroups, supporting the central role of the PI3K pathway in endometrial tumorigenesis. Conversely, *TP53* alterations were strongly associated with the p53abn subgroup, while *POLE* mutations defined the *POLE*-mutated subgroup.

In p53abn tumors, additional genomic events were less frequent and more heterogeneous, involving *PTEN*, *PIK3CA*, *SMARCA2*, *ERBB2*, and *SMARCA4* in a minority of cases. Notably, *ARID1A*, *ARID1B*, and *CTNNB1* alterations were not detected in the p53abn subgroup. This finding supports the concept that p53abn carcinomas represent a biologically distinct category, largely driven by *TP53* dysfunction and less frequently characterized by the canonical molecular alterations commonly observed in endometrioid-type tumors. The occasional identification of *ERBB2* alterations in this subgroup is also potentially relevant, considering the growing therapeutic interest in HER2-directed strategies for selected high-grade endometrial carcinomas.

*POLE*-mutant tumors exhibited the highest density of concomitant genomic alterations, consistent with their ultramutant phenotype. In addition to pathogenic *POLE* alterations, these tumors frequently harbored co-alterations in *PTEN*, *PIK3CA*, and *ARID1A*, along with additional mutations in genes including *FGFR2*, *SMARCA4*, *SMARCB1*, *ERBB2*, *ESR1*, *FGFR1*, *KRAS*, *POLD1*, and *TP53*. This high number of co-occurring genomic events should be interpreted in the biological context of defective proofreading activity, in which many additional mutations may represent passenger events rather than independent drivers of aggressive behavior. This point is clinically important because *POLE*-mutated endometrial carcinomas generally retain a favorable prognosis despite multiple additional alterations, including mutations that might otherwise be considered adverse in other molecular contexts.

The MMRd subgroup exhibited a broad, dispersed mutational spectrum. *PTEN*, *ARID1A*, and *PIK3CA* were frequently altered, and *ARID1B* alterations were relatively enriched in this group. This pattern is consistent with the hypermutated nature of mismatch repair-deficient tumors, in which defective DNA repair leads to the accumulation of heterogeneous genomic alterations across multiple pathways. The frequent involvement of chromatin-remodeling genes, including *ARID1A* and *ARID1B*, may be biologically relevant, as these genes are implicated in SWI/SNF complex function and in endometrial tumorigenesis. However, the clinical significance of these alterations within MMRd tumors remains to be clarified in larger cohorts with outcome data.

The NSMP subgroup emerged as the most heterogeneous molecular category. In this group, the most frequent alterations involved *PTEN*, *ARID1A*, *PIK3CA*, *CTNNB1*, and *KRAS*. These findings reinforce the concept that NSMP is not a single biological entity but rather a residual category that includes tumors lacking *POLE* mutations, mismatch repair deficiency, and abnormal p53 expression. Among non-class-defining alterations, *CTNNB1* was the most strikingly enriched in NSMP tumors, being detected in a substantial proportion of cases and only rarely observed outside this subgroup. This observation is particularly relevant because *CTNNB1* mutations have been proposed as potential markers of adverse outcome in otherwise low- or intermediate-risk endometrioid carcinomas. Therefore, the identification of *CTNNB1* alterations may help refine risk stratification within NSMP tumors, a group in which additional biomarkers are urgently needed to improve prognostic precision.

The relationship between histotype and molecular subgroup further supports the need for integrated morpho-molecular classification. In our cohort, serous carcinomas and carcinosarcomas were consistently MMR-proficient and assigned to the p53abn molecular subgroup, consistent with the well-established association between high-grade non-endometrioid morphology, *TP53* abnormalities, and copy-number-high biology. This finding confirms that in these histotypes, morphology and molecular classification are often concordant and that they identify tumors with aggressive biological features.

Conversely, endometrioid carcinomas were distributed across all four molecular subgroups, highlighting the marked biological heterogeneity underlying this histotype. This observation is clinically relevant, as tumors with similar endometrioid morphology may belong to molecular classes with substantially different prognostic implications, ranging from *POLE*-mutated tumors with highly favorable outcomes to p53abn tumors with adverse clinical behavior. Therefore, histotype alone is insufficient to capture the biological diversity of endometrioid endometrial carcinoma.

Dedifferentiated/undifferentiated carcinomas and clear cell carcinomas also showed molecular heterogeneity. Dedifferentiated/undifferentiated carcinomas were represented among MMRd, p53abn, and NSMP tumors, supporting the concept that this morphological category may arise in different molecular backgrounds. Similarly, clear cell carcinomas were not restricted to a single molecular class, being observed among MMRd, *POLE*-mutated, and NSMP tumors. This reinforces the notion that high-grade and morphologically ambiguous histotypes require molecular characterization to avoid over-reliance on morphology alone. Taken together, these findings emphasize that molecular classification does not replace histopathological assessment, but rather complements it. While some histotypes, such as serous carcinoma and carcinosarcoma, showed a close association with the p53abn subgroup, other categories, particularly endometrioid, clear cell, and dedifferentiated/undifferentiated carcinomas, displayed substantial molecular diversity.

Among non-class-defining alterations, *PTEN* and *PIK3CA* were broadly distributed across all molecular subgroups and therefore did not show clear class specificity. Nevertheless, their high frequency highlights the central role of PI3K/AKT/mTOR pathway deregulation in the biology of endometrial carcinoma. From a translational perspective, these alterations may become increasingly relevant as targeted therapeutic strategies directed against this pathway continue to evolve. Similarly, *KRAS* mutations, although less frequent, were identified across different molecular subgroups and may represent an additional layer of biological and therapeutic heterogeneity.

Beyond alteration frequencies, the distribution of recurrent hotspots differed across molecular subgroups (Table 3 and Table S1). All *CTNNB1* alterations affected exon 3 and were almost exclusively confined to the NSMP subgroup (a single *POLE*-mutated case carried a non-canonical exon 3 variant), clustering at the GSK3β-phosphorylation/degron codons Asp32, Ser33, Gly34, and Ser37 (e.g., p.S33A, p.S33F, p.S37F, p.G34V, p.D32N), consistent with canonical Wnt-pathway activation and with the proposed adverse prognostic role of *CTNNB1*-mutated NSMP tumors. *PIK3CA* alterations were distributed across all subgroups and involved both the helical (p.E542K, p.E545A/D) and kinase (p.H1047R/Y) domains, in line with the central role of PI3K/AKT/mTOR signaling. *KRAS* alterations recurrently affected codons 12 and 13 (p.G12D, p.G12V, p.G12A, p.G13C, p.G13D) across subgroups. In the p53abn subgroup, *TP53* alterations predominantly involved DNA-binding-domain hotspots (e.g., p.R248Q, p.R273C/G/H, p.R283H), whereas *PTEN* alterations were largely truncating or frameshift, with a recurrent hotspot at R130 (p.R130Q/G/*), consistent with loss of function. Although the limited subgroup sizes preclude firm conclusions, these observations indicate that hotspot-level analysis—rather than gene-level frequency alone—refines the biological interpretation of endometrial carcinoma subgroups and highlights potential therapeutic vulnerabilities, particularly PI3K-pathway and Wnt-pathway targeting.

A limited number of alterations appeared restricted to specific molecular settings. Excluding the class-defining genes *TP53* and *POLE*, alterations in *SMARCB1*, *ESR1*, and *SLFN11* were observed exclusively in *POLE*-mutated tumors. However, these findings should be interpreted with caution because of the small number of *POLE*-mutated cases and the high background mutational burden typical of this subgroup. Rather than indicating true subgroup specificity, these alterations may reflect the accumulation of passenger mutations in ultramutated tumors. Larger studies are needed to determine whether any of these events have biological or clinical relevance.

Another relevant aspect of this study is the comparison between immunohistochemical and NGS findings. For p53, discordance between IHC and *TP53* mutational status was observed in only a small subset of cases. This is not unexpected, as some *TP53* mutations may not result in a clearly abnormal immunohistochemical pattern, while abnormal p53 expression may occasionally occur in the absence of detectable *TP53* mutations using targeted sequencing. Possible explanations include alterations outside the analyzed regions, technical limitations, tumor heterogeneity, or alternative mechanisms affecting protein expression. For *ARID1A* and *PTEN*, discordances between IHC and NGS were more frequent. These discrepancies may reflect several factors, including subclonal alterations, copy-number changes, epigenetic silencing, mutations not covered or not detected by the panel, protein degradation, or interpretative issues related to heterogeneous staining. These findings emphasize that IHC and NGS provide complementary, rather than interchangeable, information.

Comprehensive studies based on large targeted panels have extensively characterized the genomic landscape of endometrial carcinoma [16,17]. Our work is complementary to these efforts: rather than expanding molecular subclassification, it validates a focused, cost-effective panel tailored to routine FFPE diagnostics, aimed at delivering reliable and actionable molecular information within existing clinical workflows. The use of an extended-customized NGS panel offers several practical advantages. First, it enables identification of *POLE* mutations, which cannot be assessed by immunohistochemistry and are essential for accurate molecular classification. Second, it can detect multiple molecular alterations in a single assay, optimizing tissue use and reducing the need for sequential testing. Third, it provides information on potentially actionable alterations that may become clinically relevant in advanced, recurrent, or treatment-resistant disease. This is particularly important in the current era of precision oncology, in which molecular features increasingly guide therapeutic decisions. This panel was designed for somatic molecular profiling of tumor tissue and not for the detection of germline variants. Accordingly, the identification of an inherited cancer predisposition, such as Lynch syndrome, is not an objective of this assay and is addressed through dedicated reflex germline testing performed within a distinct diagnostic pathway by medical genetics specialists. Somatic tumor profiling and germline risk assessment therefore represent complementary but separate processes.

From a performance standpoint, the analytical validation of the sequencing chemistry underlying this panel—including sensitivity, specificity, and reproducibility for the detection of single-nucleotide variants and small insertions/deletions in FFPE-derived DNA—has been established in a previous dedicated study [18]. In the present cohort, analytical quality was confirmed by a mean target coverage exceeding 1000×, bidirectional variant confirmation, and a 5% variant-allele-frequency calling threshold. Clinically, the panel reliably enabled assignment of the WHO/ESGO-ESTRO-ESP molecular class in all evaluable cases, identifying *POLE*-mutated and p53-abnormal tumors by sequencing and complementing MMR immunohistochemistry. Thus, although the panel is not intended to reproduce the breadth of comprehensive commercial platforms, it delivers the molecular information required for guideline-based classification with performance suitable for routine diagnostic use.

This study has some limitations. The cohort size was relatively limited, particularly for the *POLE*-mutated subgroup, which limits the interpretation of subgroup-specific or apparently exclusive alterations. The correlation between histological subtype and molecular subgroup should be regarded as descriptive and hypothesis-generating rather than definitive. Several histotype categories were represented by only a few cases, and the cohort was intentionally designed to span the full spectrum of endometrial carcinoma encountered in routine diagnostic practice. Consequently, subtype-specific molecular observations require confirmation in larger and more homogeneous series—for example, cohorts restricted to MMR-proficient endometrioid carcinomas.

Moreover, consistent with its primary aim—the analytical and clinical validation of the NGS panel—the present analysis was descriptive and was not designed to correlate the identified molecular alterations with clinicopathological features or patient outcome. Although such correlations are highly relevant, they were beyond the scope of the present work and will be addressed in future studies on larger, outcome-annotated cohorts.

The inclusion of variants of uncertain significance may also influence the apparent frequency of altered genes, although benign and likely benign variants were excluded. Finally, targeted sequencing does not capture all possible mechanisms of gene inactivation, including promoter methylation, large structural variants, copy-number alterations, or epigenetic modifications, which may partly explain discordances with immunohistochemistry.

## 4. Materials and Methods

### 4.1. Study Cohort and Clinicopathologic Parameters

After approval by the local ethics committee CE-AVEC (Comitato Etico Area Vasta Emilia Centro, registration nos. 27/2019/Sper/AOUBo and 10/2023/Sper/AOUBo), all 208 enrolled patients signed informed consent before surgical resection and diagnosis. Surgical hysterectomy and staging were performed at the Division of Gynecologic Oncology, “IRCCS Azienda Ospedaliero-Universitaria di Bologna” (Bologna, Italy). For each case, Formalin-Fixed Paraffin-Embedded (FFPE) representative blocks were obtained from the Pathology Unit’s files by an expert pathologist (A.D.L.). All immunohistochemical (IHC) and molecular analyses were performed on whole tissue sections.

A total of 70 cases of primary endometrial carcinoma were randomly selected for the analysis. For each case, a representative FFPE tissue block was retrieved from the archives of the Anatomic Pathology Unit of “IRCCS Azienda Ospedaliero-Universitaria di Bologna” (Bologna, Italy) and was used for molecular and IHC analyses at the Solid Tumor Molecular Pathology Laboratory, IRCCS Azienda Ospedaliero-Universitaria Di Bologna (Bologna, Italy).

### 4.2. Immunohistochemistry

3 µm serial sections were cut from each FFPE block and processed using an automated Benchmark Ultra immunostainer (Ventana Medical Systems, Tucson, AZ, USA). Immunohistochemical staining for ARID1A, β-catenin, p53, PTEN, MLH1, PMS2, MSH2, and MSH6 was performed using Ventana antibodies and the OptiView DAB detection kit Ventana Diagnostic Systems, Tucson, AZ, USA, with brown chromogenic visualization. Sections were counterstained with Hematoxylin and Bluing reagent according to the manufacturer’s instructions.

p53 expression was considered abnormal/mutant-like p53abn in the presence of one of the following staining patterns: (i) strong diffuse nuclear overexpression in more than 50% of tumor cells; (ii) complete absence of nuclear staining, null pattern in tumor cells, in the presence of an appropriate internal positive control; or (iii) aberrant cytoplasmic staining. All other staining patterns were interpreted as wild-type.

PTEN expression was classified as positive when uniform or heterogeneous cytoplasmic and/or nuclear staining was observed in neoplastic cells, and as negative when complete loss of cytoplasmic and nuclear staining was present in tumor cells, with positive internal controls.

Mismatch repair MMR protein expression was evaluated for MLH1, PMS2, MSH2, and MSH6. Each protein was scored as lost when a complete absence of nuclear staining was observed in tumor cells, with retained nuclear staining in internal control cells. Loss of expression of at least one of the four MMR proteins, or combined loss of MLH1/PMS2 or MSH2/MSH6, was interpreted as a mismatch repair deficiency (MMRd).

ARID1A was considered lost when a complete absence of nuclear staining was observed in tumor cells, in the presence of retained staining in stromal or inflammatory internal control cells. β-catenin expression was assessed by evaluating membranous and nuclear staining; cases showing nuclear β-catenin accumulation were considered abnormal. Ki67 proliferative activity was assessed as the percentage of positively stained tumor cell nuclei.

### 4.3. DNA Extraction and Next-Generation Sequencing

DNA was extracted from two to three 10 µm thick sections using the QuickExtract FFPE DNA Extraction Kit (Epicenter, Madison, WI, USA) by scraping the area of interest according to the selection by a pathologist on the final Hematoxylin and Eosin (H&E) section. DNA concentration was measured using a Qubit fluorometer (Thermo Fisher Scientific, Waltham, MA, USA) with the Qubit 1X dsDNA BR Assay Kit (Thermo Fisher Scientific), following the manufacturer’s instructions. NGS analysis was performed using a multi-gene panel developed in the Solid Tumor Molecular Pathology Laboratory of IRCCS Policlinico di S.Orsola. The panel allows the analysis of the following coding sequencing and hot-spot regions of 28 genes for a total of 1040 amplicons (human reference sequence hg19/GRCh37) in the following genes: *ACTL6A* (CDS), *ACTL6B* (CDS), *ARID1A* (CDS), *ARID1B* (CDS), *BRAF* (Exons 11, 15), *CTNNB1* (Exons 3, 7, 8), *ERBB2* (CDS), *ESR1* (CDS), *FGFR1* (CDS), *FGFR2* (CDS), *FGFR3* (CDS), *FGFR4* (CDS), *FOLH1* (CDS), *HRAS* (Exons 2–4), *KRAS* (Exons 2–4), *NRAS* (Exons 2–4), *PIK3CA* (CDS), *POLD1* (CDS), *POLE* (CDS), *PPP2R1A* (CDS), *PTEN* (CDS), *SLFN11* (CDS), *SMARCA2* (CDS), *SMARCA4* (CDS), *SMARCB1* (CDS), *SPOP* (CDS), *TERT* (promoter), *TP53* (CDS).

NGS was performed using the Gene Studio S5 Prime Sequencer (Thermo Fisher Scientific, Waltham, MA, USA). For amplicon library preparation, the AmpliSeq Plus Library Kit was used. Templates were prepared with an Ion Chef Machine and sequenced with an Ion 530 chip on the S5 Prime Sequencer (Thermo Fisher Scientific, Waltham, MA, USA). Sequences were analyzed using the Ion Reporter tool (v.5.20; Thermo Fisher Scientific, Waltham, MA, USA). The sequencing reads passed the quality control assessment, as the coverage for each target region exceeded 1000×. Filtered variants were then manually examined, and only nucleotide variations discovered in both strands and at least 5% of the total number of reads analyzed were considered for the mutational calls [6,18]. The pathogenicity of each mutation was assessed using the Varsome tool (https://varsome.com/, last accessed on 31 March 2026). Benign/likely benign variants were not considered in the present study. All data used in the present study were completely anonymized and aggregated.

### 4.4. Molecular Classification of Endometrial Carcinoma

Cases were classified as *POLE*, MMRd, NSMP, and p53abn according to the WHO algorithm. First, only *POLE* pathogenic variants were used to assign the *POLE* subtype; then, consecutive IHC analysis for MMR proteins and p53 expression was evaluated to define MMRd and p53abn tumors. Tumors with normal MMR and p53 expression and no *POLE* mutations were classified as NSMP [6,19].

## 5. Conclusions

The present study validates a focused, laboratory-developed NGS panel as a practical and affordable tool for the molecular classification of endometrial carcinoma in routine clinical practice, providing the information required for guideline-based subgrouping without the cost and logistical demands of comprehensive commercial platforms. In conclusion, our results support the clinical and biological value of an extended NGS approach in endometrial carcinoma. Beyond identifying *POLE* and *TP53* alterations required for molecular classification, a customized multi-gene panel revealed additional genomic features that differ across molecular subgroups. In particular, *CTNNB1* alterations were enriched in NSMP tumors, suggesting a potential role in further stratifying this heterogeneous category. *POLE*-mutated tumors showed the highest burden of concomitant alterations, consistent with their ultramutated phenotype, whereas p53abn tumors were largely dominated by *TP53* abnormalities. The correlation between histotype and molecular subgroup further highlights the importance of an integrated diagnostic approach, especially for endometrioid, clear cell, and dedifferentiated/undifferentiated carcinomas, which may show substantial molecular diversity. Overall, expanded molecular profiling may complement current surrogate classification and contribute to more refined biological and, potentially, therapeutic stratification of endometrial carcinoma.

## Figures and Tables

**Table 1:** Comparison of the laboratory-developed panel used in this study with representative comprehensive commercial genomic profiling assays.

Feature	In-House Panel (This Study)	FoundationOne CDx	MSK-IMPACT	TSO500
Number of Genes	28 (targeted for EC classification)	324	up to ~505	523
Variant classes/biomarkers	SNVs, indels (CDS/hotspot regions)	SNVs, indels, CNAs, fusions; TMB, MSI	SNVs, indels, CNAs, rearrangements; MSI, TMB	SNVs, indels, CNVs, fusions; TMB, MSI
Sample/DNA input	FFPE; ~10–30 ng	FFPE, NA	FFPE; matched tumour + normal, 100–250 ng	FFPE; ~30–40 ng
Setting/turnaround	In-house; short turnaround	Centralized/send-out	Single-site (MSKCC); In-house	In-house or service laboratory
Intended use	Molecular classification of EC in daily routine practice	Pan-cancer CGP	Pan-cancer CGP	Pan-cancer CGP

FFPE, Formalin-fixed and Paraffin-Embedded; CGP, comprehensive genomic profiling; EC, endometrial carcinoma.

**Table 2:** Clinicopathologic characteristics and molecular subgroups of the analyzed cohort.

Characteristics of EC Cases	*n* = 70 (%)
Age, years	66.1
	(37–91)
Histotype	
Endometrioid	54 (77.1%)
Dedifferentiated/Undifferentiated	6 (8.6%)
Clear cell	4 (5.7%)
Serous	3 (4.3%)
Carcinosarcoma	3 (4.3%)
MMR status	
MMRp	53 (75.7%)
MMRd	17 (24.3%)
Protein loss ^	
MLH1/PMS2	15 (88%)
MSH2/MSH6	3 (18%)
Molecular subgroup	
MMRd	17 (24.3%)
p53abn	16 (22.9%)
POLE	9 (12.9%)
NSMP	28 (40.0%)

^ In one case, all four MMR proteins were lost.

**Table 3:** Summary of recurrent hotspots by molecular subgroup in the present cohort. * Indicate a stop codon (Ter).

Gene	Enriched Subgroup	Recurrent Hotspots (This Cohort)	Implication
*CTNNB1*	NSMP (almost exclusive)	Exon 3: D32N, S33A/F, G34V, S37F	Wnt-pathway activation; candidate adverse marker in NSMP
*PIK3CA*	All subgroups	Helical domain E542K, E545A/D; kinase domain H1047R/Y	PI3K/AKT/mTOR signaling; potential target
*KRAS*	All subgroups	Exon 2, Codons 12/13: G12D/V/A, G13C/D	RAS/MAPK signaling
*TP53*	p53abn	DNA-binding domain: R248Q, R273C/G/H, R283H	Class-defining; loss of p53 function
*PTEN*	All subgroups	Predominantly truncating/frameshift; hotspot R130 (Q/G/*)	Loss of function; PI3K-pathway activation

## Data Availability

The original contributions presented in this study are included in the article/Supplementary Materials. Further inquiries can be directed to the corresponding author.

## Supplementary Materials

The following supporting information can be downloaded at https://www.mdpi.com/article/10.3390/ijms27167384/s1.
